# Membrane-Active Properties of an Amphitropic Peptide from the CyaA Toxin Translocation Region

**DOI:** 10.3390/toxins9110369

**Published:** 2017-11-14

**Authors:** Alexis Voegele, Orso Subrini, Nicolas Sapay, Daniel Ladant, Alexandre Chenal

**Affiliations:** 1 Département de Biologie Structurale et Chimie, Institut Pasteur, Unité de Biochimie des Interactions Macromoléculaires, CNRS UMR 3528, 28 Rue du Dr Roux, 75724 Paris, CEDEX 15, France; alexis.voegele@outlook.fr (A.V.); orso.subrini@gmail.com (O.S.); 2 Université Paris Diderot Paris VII, 75013 Paris, France; 3 Institut de Biologie Structurale, 71 Avenue des Martyrs, 38044 Grenoble, CEDEX 9, France; 4 Bioaster Technology Research Institute, 69007 Lyon, France; nicolas.sapay@bioaster.org

**Keywords:** membrane-active peptide, adenylate cyclase, CyaA toxin, repeat in toxin, membrane partitioning-folding coupling, membrane disruption, arginine side chain

## Abstract

The adenylate cyclase toxin CyaA is involved in the early stages of infection by *Bordetella pertussis*, the causative agent of whooping cough. CyaA intoxicates target cells by a direct translocation of its catalytic domain (AC) across the plasma membrane and produces supraphysiological levels of cAMP, leading to cell death. The molecular process of AC translocation remains largely unknown, however. We have previously shown that deletion of residues 375–485 of CyaA selectively abrogates AC translocation into eukaryotic cells. We further identified within this “translocation region” (TR), P454 (residues 454–484), a peptide that exhibits membrane-active properties, i.e., is able to bind and permeabilize lipid vesicles. Here, we analyze various sequences from CyaA predicted to be amphipatic and show that although several of these peptides can bind membranes and adopt a helical conformation, only the P454 peptide is able to permeabilize membranes. We further characterize the contributions of the two arginine residues of P454 to membrane partitioning and permeabilization by analyzing the peptide variants in which these residues are substituted by different amino acids (e.g., A, K, Q, and E). Our data shows that both arginine residues significantly contribute, although diversely, to the membrane-active properties of P454, i.e., interactions with both neutral and anionic lipids, helix formation in membranes, and disruption of lipid bilayer integrity. These results are discussed in the context of the translocation process of the full-length CyaA toxin.

## 1. Introduction

The adenylate cyclase toxin (CyaA or ACT) is one of the major virulence factors produced by *Bordetella pertussis*, the causative agent of whooping cough [1,2]. CyaA plays a crucial role in the early stages of respiratory tract colonization by *B. pertussis* [3]. CyaA is a 1706-residue long multi-functional, and multi-domain protein [4,5,6,7], composed of a catalytic domain (AC, residues 1–364) [8,9,10], a translocation region (TR, approx. 370–500) [10,11], a hydrophobic region (HR, residues 500–750), an acylation region (AR, residues 750–1000), and a C-terminal Repeats-in-ToXin (RTX) calcium-binding domain (RD, residues 1000–1706). After synthesis, CyaA is acylated intra-cellularly by the CyaC transacylase and secreted in unfolded conformations through a dedicated type 1 secretion system. Upon secretion, calcium binding to the RTX motifs triggers the cooperative folding of the RD domain followed by the whole CyaA toxin [12,13,14,15,16,17,18,19,20,21]. Indeed, calcium is an essential cofactor of CyaA cytotoxic activities [5,6,7]. After binding to its cell receptor CD11b/CD18 [22,23,24,25], or upon direct interaction with the membrane, CyaA inserts its hydrophobic region HR into the plasma membrane of eukaryotic target cells. The AC domain is then directly translocated across the plasma membrane into the cytosol of the target cells [4,6,26], where it is activated by calmodulin to produce supra-physiological levels of cAMP. Accumulation of cAMP alters cell physiology and may ultimately lead to cell death. Besides this, as with many other RTX cytolysins, CyaA is endowed with a pore-forming activity, which is able to induce cell lysis, an activity that is usually monitored by hemolysis of erythrocytes [27,28,29,30]. 

The mechanisms by which the AC of CyaA is translocated across the plasma membrane of eukaryotic target cells still remain largely unknown at the molecular level. The translocation process, however, is known to be dependent on several parameters, including the presence of free calcium in the extracellular medium [7,22], the CyaC-mediated post-translational acylation of CyaA on two lysines (K860 and K983) [31,32,33,34,35], the membrane potential [36,37], and temperature (required for membrane fluidity) [22]. Besides, it was recently suggested that CyaA may be endowed with a phospholipase activity that seems to be required for the AC domain translocation [26].

We have previously shown that the translocation region (TR), encompassing residues 384–489 of CyaA, is essential for transport of the AC domain across the cell membrane. We demonstrated that the deletion of TR selectively abrogated the ability of CyaA to trigger cAMP accumulation in target cells, while it did not affect CyaA cell binding nor its hemolytic activity [10]. Furthermore, while the AC domain alone cannot bind membranes, a longer polypeptide, AC489 (residues 1 to 489 of CyaA), encompassing AC and TR, is able to bind and permeabilize membranes. We subsequently identified within TR a polypeptide segment (residues 454 to 484), which exhibits membrane-active properties [11]. The peptide corresponding to this sequence, called P454, partitions into membranes, acquires a helical conformation, and induces lipid bilayer permeabilization. We proposed that this region may locally destabilize the lipid bilayer and thus favor AC translocation across the plasma membrane [11]. Recently, Masin et al. reported that most of the TR region from residues 411 to 490 is inserted into membrane [30]. They further showed that substitutions of arginine residues in the TR region decreased the translocation efficiency of CyaA, while neutralization of negatively charged aspartate and glutamate residues affected CyaA pore-forming activity [30]. 

In the present article, we characterize the membrane interacting properties of various predicted amphitropic regions of CyaA. Amphitropic regions are involved in the regulation of both the folding and functions of peripheral proteins and membrane proteins [38,39]. We show that several of these amphitropic CyaA peptides are able to interact with membranes and fold upon membrane interaction. However, only P454 is able to permeabilize membranes. We further analyze the contribution of the arginine residues of P454, R461, and R474, and characterize their involvement in lipid bilayer insertion, membrane-induced secondary structure formation, and membrane permeabilization. We show that P454 does exhibit several characteristics of antimicrobial peptides, in particular, the contribution of arginine residues to membrane-active properties, such as folding upon membrane insertion and disruption of the lipid bilayer packing. We discuss these results in the context of the CyaA toxin.

## 2. Results

### 2.1. Experimental Determination of Membrane Partition of CyaA-Derived Peptides

**Figure 1:**
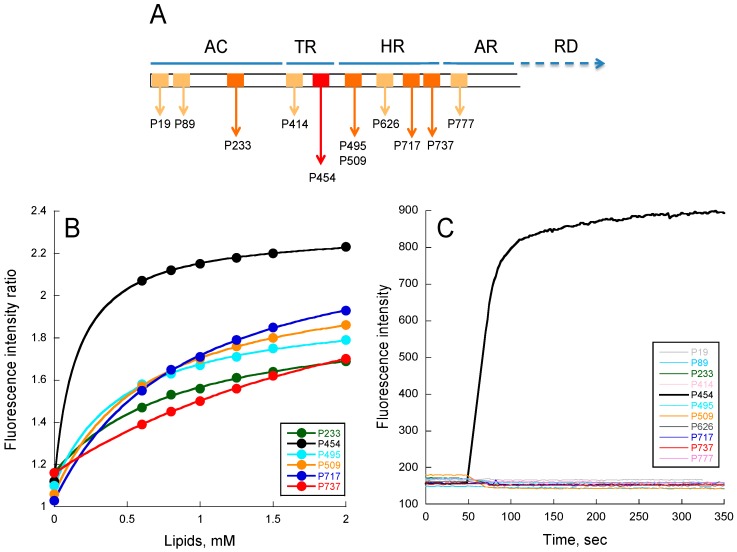
Peptide membrane partition followed by tryptophan intrinsic fluorescence. (**A**) Localization of CyaA-derived peptides on the full-length toxin. Each box represents a peptide. Light orange boxes correspond to peptides unable to interact with membranes (P19, P89, P414, P626, and P737); orange boxes (P233, P495, P509, P717, and P737) correspond to peptides able to partition into membranes but that do not permeabilize membranes; the red box corresponds to the unique P454 peptide able to interact and permeabilize membranes; (**B**) Ratio of fluorescence intensity (340 nm/380 nm, excitation at 280 nm) as a function of lipid concentration. The negatively charged small unilamellar vesicles (SUVs) were made of dashed blue (DOPC/DOPE/DOPG/Chol at ratio 4:3:2:1); (**C**) Membrane permeabilization assay. Time course of fluorophore probe (ANTS) and quencher (DPX) efflux (excitation at 390 nm and emission at 515 nm) from negatively charged LUVs (0.45 mM lipids) were prepared at a molar ratio DOPC/DOPE/DOPG/Chol 4:3:2:1. Experiments were performed in the presence of 1 μM of P454 (black) and 10 μM for all other CyaA-derived peptides. Standard deviations (S.D.) are within the size of the dots.

To characterize the properties of predicted membrane-active regions of CyaA, 11 peptides were produced by peptide synthesis, corresponding to short sequences exhibiting amphiphilic properties identified in silico and with hydrophobicity, and hydrophobic moments located within the Surface-Membrane area of the <H>-μH plot of Eisenberg et al. [40]. The properties of these peptides (designated by the residue number of their N-terminal extremity in Figure 1A) are described in ‘’Materials and Methods’’ and Tables S1 and S2 and Figure S1.

We have analyzed their ability to (i) partition from the solution to the membrane (SUVs made of DOPC/DOPE/DOPG/Chol 4:3:2:1, i.e., the lipid composition used in our previous studies [10,11]) by tryptophan intrinsic fluorescence; (ii) fold upon membrane partitioning by far-UV circular dichroism; and (iii) induce a local destabilization of lipid bilayers as monitored by permeabilization assay. We have further investigated these peptides by molecular dynamics to get more insights into their conformations in membranes.

**Figure 2:**
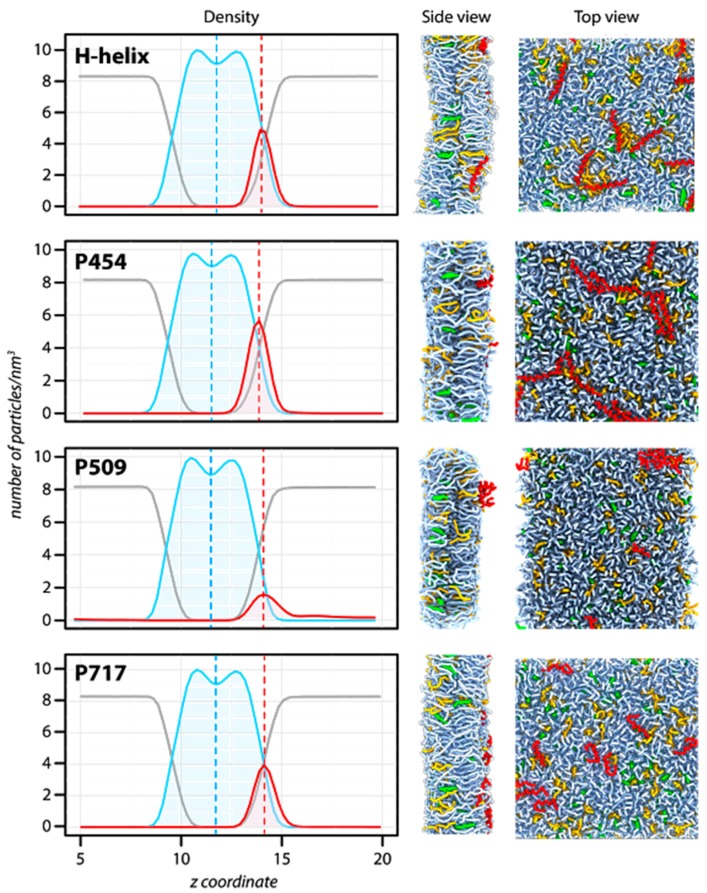
Molecular dynamics of CyaA-derived peptides in the presence of membrane. Summary of the molecular dynamics simulations in the presence of dashed green (DOPG), including the particle density along the trans-membrane axis (**left**), the side (**middle**), and the top (**right**) views of the systems after 1 μs of simulation. *Left panel*: The density is calculated for water (grey), lipids (blue), and peptides (red). The center of the bilayer is indicated by a blue dashed line. The average position of each peptide is indicated by a dashed red line. The peptide density is four times magnified to better visualize the position of each peptide. *Middle and right panels*: Snapshots of the systems after 1 μs of molecular dynamics simulation at constant temperature (323.15 K) and pressure (1 bar). The peptides are depicted in red, DOPC molecules in light blue, cholesterol molecules in green, and DOPG molecules in orange. For clarity, water is not depicted.

From tryptophan fluorescence and circular dichroism (CD) data (Figure 1B and Figure S2 respectively), we found that the following peptides were able to interact with membranes: P233, P454, P495, P509, P717, and P737. For P19, P89, P414, P626, and P777 peptides, no detectable change of tryptophan fluorescence or CD signature was observed upon addition of membranes (data not shown). The six peptides interacting with membranes display various free energy (ΔG) values calculated from their partition coefficients (K_X_, see Table S3), obtained from fluorescence titrations (see Material and Methods for details). They also exhibit various membrane-induced secondary structure changes: the P454, P717, and P233 peptides show a higher helical content than P737, P495, and P509. The low alpha-helical content of P737 is likely related to its low K_X_, while P495 and P509, which both exhibit relatively high K_X_, are likely to adopt non-canonical structures in membranes, as suggested by far-UV CD and molecular dynamics (see Figure 2).

### 2.2. Molecular Dynamics of CyaA-Derived Peptides in Lipid Bilayers

We performed molecular dynamics simulations of DOPC/DOPG/Chol 8:1:1 lipid mixture with the following peptides: P233 (H-helix), P454, P509, and P717. Snapshots of the systems after 1 μs of molecular dynamics simulation are shown in Figure 2 to illustrate the general organization of the peptide/lipid mixtures following the simulations. To better characterize the peptide partition in the bilayer, the density of lipids, peptides, and water was determined along the transmembrane axis *z* (Figure 2, left panels). The size of the bilayer, corresponding to the width of the lipid density, is almost identical in all the simulated systems (peak-to-peak distance ≈ 2.0 nm, corresponding to the approximate acyl chain thickness), as well as the lipid bilayer hydration. The position of the peptide density significantly changes between systems, however. P509 is located on average at 2.6 nm from the bilayer center, while P233, P454, and P717 are located at 2.2, 2.35, and 2.4 nm, respectively. Therefore, P233, P454, and P717 are more embedded in the bilayer than P509. Notably, P233, P454, and P717 exhibit regular helical conformations, while P509 adopts non-canonical conformations, as observed on the system top view (Figure 2), and as suggested by circular dichroism data (Figure S2). These observations highlight the diversity of conformations, depths of penetration, and thermodynamics of the CyaA-derived peptides investigated herein. 

### 2.3. Lipid Vesicle Permeabilization

We then investigated the ability of the peptides to permeabilize LUVs composed of DOPC/DOPE/DOPG/Chol 4:3:2:1 (Figure 1C). Permeabilization was monitored using the ANTS/DPX assay (see methods). As expected, peptides such as P19, P89, P414, P626, and P777, which are not able to partition into membrane, do not show any permeabilization activity, even at high peptide concentrations up to 10 μM (Figure 1C). Surprisingly, among the peptides P233, P454, P495, P509, P717, and P737, which partition into membranes, only P454 was able to permeabilize LUVs. Hence, P454 is the unique peptide from the CyaA-derived peptide series able to destabilize the lipid bilayer of LUVs, suggesting that it has a specific role in CyaA invasive activity. We therefore decided to investigate more thoroughly the properties of the P454 peptide.

### 2.4. Effects of Non-Lamellar Lipids, Charge, and Acyl Chains Fluidity on P454 Membrane Partition

We examined the effect of lipid polymorphism and charge on the partition of P454. The lipid of reference is DOPC, a neutral, lamellar-forming lipid with a cylindrical shape in liquid-disordered state at room temperature, to which we added anionic, lamellar bilayer forming DOPG lipid. We also tested the effect of neutral DOPE and cholesterol, which increase membrane rigidity and DOPC acyl chains straightening [41,42,43], as well as promote inverted hexagonal structures (H_II_) in PC lipid bilayers [44,45]. 

**Figure 3:**
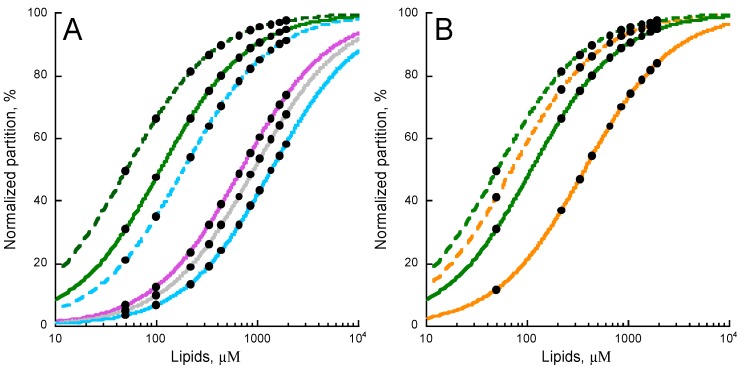
Effects of lipid composition on P454 membrane partition. (**A**) P454 membrane partition in DOPC (green), DOPC/DOPG 8:2 (dashed green), DOPC/DOPE 7:3 (purple), DOPC/Chol 7:3 (grey), DOPC/DOPE/Chol 6:3:1 (blue), and DOPC/DOPE/DOPG/Chol 4:3:2:1 (dashed blue) SUVs. (**B**) P454 membrane partition in DOPC SUVs in the absence (green) and in the presence of DOPG (dashed green); in POPC SUVs in the absence (orange) and in the presence of POPG (dashed orange). Data sets of K_X_ and ΔG values are given in Table S4. S.D. are within ±5%.

The partition of P454 into DOPC (green trace) is shown in Figure 3A. The addition of the negatively charged DOPG (green dash trace) to DOPC membranes increases the affinity of P454 for membranes. On the contrary, addition of DOPE (purple trace) or cholesterol (grey trace) or both (blue trace) to DOPC membranes progressively decreases the affinity of P454 for these membranes. The addition of DOPG (blue dash trace) to DOPC/DOPE/Chol (blue trace) partially compensates for the effects of DOPE and cholesterol.

Cholesterol and DOPE, which undergo lamellar/inverse hexagonal (Lα–H_II_) phase transition below 10 °C [46], both favor non-lamellar bilayer structures at 25 °C. This suggests that negative curvatures may be unfavorable for P454 membrane partition. PE and cholesterol also decrease the fluidity of liquid-disordered lipid bilayers [42,43], and the increased membrane rigidity may likewise disfavor P454 partitioning into membranes. 

We further investigated the effect of the hydrocarbon core fluidity on P454 membrane partition by increasing acyl chain rigidity. DOPC and DOPG lipids were substituted by POPC and POPG, which are characterized by higher phase transition temperatures. Figure 3B shows that the membrane partition of P454 is reduced in POPC membranes (orange trace) as compared to DOPC (green trace) lipid bilayers. The addition of POPG in POPC membranes (dash orange trace) does increase the membrane partition of P454, as observed for DOPC/DOPG (dash green trace). Finally, the addition of both POPE and cholesterol to POPC totally abolishes the partition of P454 (not shown), in agreement with the effects of PE and cholesterol observed with DO-phospholipids. Taken together, P454 partition is favored in the presence of lamellar-structure forming lipids, and in negatively charged membranes in the liquid-disordered state (see Table S4).

### 2.5. Membrane Partition of P454-Derived Peptides

After determining the main lipid properties favoring peptide membrane partition, we have investigated the contribution of the two native arginines of P454, R461, and R474 (hereafter referred to R1 and R2, respectively), to membrane partitioning, membrane-induced helical formation, and membrane permeabilization. The two arginines (R1, R2, or both: R12) were substituted by (i) lysines, (R12K) to preserve the positive charge, while removing the guanidinium group at the extremity of arginine side chains; (ii) glutamines (R1Q, R2Q, and R12Q), to neutralize the side chains and keep similar bulky side chains; (iii) alanines (R12A), to neutralize the side chains and reduce the bulkiness of arginine side chains, and (iv) glutamates (R12E), to reverse the charge.

**Figure 4:**
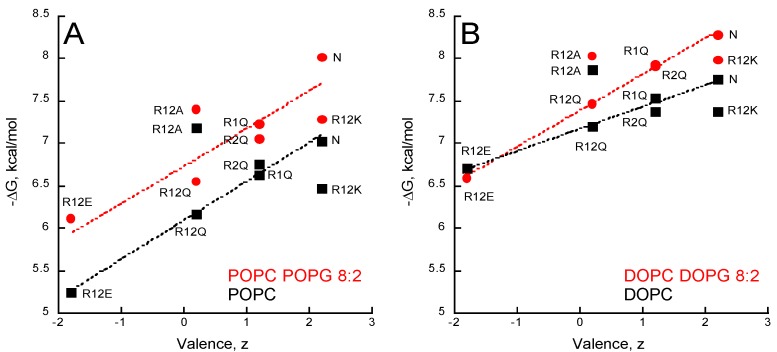
Valence-dependent solution-to-membrane partitioning of P454-derived peptides. (**A**) Free energy of membrane partitioning for peptides in POPC/POPG 8:2 (circles) and POPC (squares) SUVs. (**B**) Free energy of membrane partitioning for peptides in DOPC/DOPG 8:2 (circles) and DOPC (squares) SUVs. Solution-to-membrane partition of peptides was recorded by tryptophan intrinsic fluorescence. Titration data sets are shown in Figure S3 and were used to determine the K_X_ and ΔG values (see Table S5). The valence of peptides is given in Table S1. S.D. are ±0.1 kcal/mol.

The partition coefficients K_X_ and the ΔG values (Table S5) of the seven peptides were obtained from titration experiments followed by tryptophan fluorescence (Figure S3). Figure 4 shows the ΔG values reported as a function of the peptide valence, z, in the presence of POPC ± POPG (Figure 4A) and DOPC ± DOPG (Figure 4B). A correlation between the peptide valence and the ΔG of membrane partition is observed. Linear regressions (red and black dash lines in Figure 4) show that membrane partition of peptides is favored in the presence of membranes containing anionic PG lipids (red lines vs black lines) and in the more fluidic DO-phospholipids (Figure 4B) than in the PO-phospholipids (Figure 4A). However, no correlation was observed between the hydrophobic moment, the mean hydrophobicity, and free energy of membrane partition (ΔG) among the P454 peptide series (Figure S4), indicating that their amphiphilicity is not a primary criterion for membrane affinity.

Arginine substitutions significantly affect the ∆G of partition, with ∆G ranging from −5 to −8 kcal/mol. The peptides follow a ∆G-valence dependency from the negatively charged R12E (z = −1.8 e) to the native (N) P454 peptide (z = +2.2 e). The three peptides with arginine to glutamine substitutions (R1Q, R2Q, and R12Q) nicely follow the ∆G-valence dependency, providing further evidence that membrane partitioning correlates with peptide charge (Figure 4A,B).

The membrane partition of R12A depends on the charge of the membrane. R12A is slightly more efficient than WT P454 to partition into neutral DOPC and POPC membranes, while in negatively charged membranes (DOPC/DOPG and POPC/POPG) its ∆G values are lower than those measured for the native P454 peptide. This further highlights the contribution of electrostatic interactions between P454 and anionic membranes. Notably, the R12A peptide has the same valence as R12Q (z = +0.2 e). The R12A ∆G values are higher than R12Q ∆Gs for all membrane lipid compositions tested, suggesting that the substitution from glutamine to alanine favors membrane interaction, in agreement with the White-Wimley interfacial scale [47].

The White-Wimley scales predict [47] that substitutions of arginine to glutamine or alanine should significantly favor membrane binding, but our experimental data shows that R1Q, R2Q, R12Q, and R12A have a reduced affinity for membranes (or slightly improved for R12A in neutral membranes) compared to WT P454. This observation supports the view that arginine favors membrane partitioning, in agreement with recent data on arginine in membranes [48].

The R12K ∆G values are lower than those of the native P454 peptide for all membrane lipid compositions tested, indicating that, for the same valence, arginine residues provide a significant net contribution of about ∆∆G = 0.5 ± 0.2 kcal/mol to the partition compared to the lysine residues of R12K. From a thermodynamic perspective, this ΔΔG fits nicely with the White-Wimley interfacial scale: the ∆G from water to interface is 0.81 and 0.99 kcal/mol for arginine and lysine, respectively, and 0.4 kcal/mol for two substitutions. From a structural point of view, this is likely due to the guanidinium-phosphate bidentate complex establishing hydrogen bonds between the guanidinium group of arginine side chains and the phosphate group of phospholipids (see discussion). To summarize, this data suggests that hydrophobic forces, attractive electrostatic interactions, and hydrogen bonds contribute to P454 membrane insertion.

### 2.6. Synchrotron Radiation Circular Dichroism of P454-Derived Peptides

Secondary structure content changes of P454-derived peptides upon membrane partition were studied in the far-UV range (Figure S5) by synchrotron radiation circular dichroism (SRCD). CD spectra were deconvoluted using BestSel [49] to estimate the alpha helical content in each experimental condition (Table S6 and Figure S6). The data shows that P454 peptides are mainly characterized by structural disorder in solution, with the exception of R12A, which exhibits a slightly higher helical content than the native P454 peptide in solution. The structural disorder was evidenced by the presence of a negative π-π band (190–210 nm) and the low intensity of the n-π band (210–230 nm). The addition of TFE induced a large increase of helical content for all peptides, as evidenced by the splitted π-π band and the strong negative n-π band (Figure S5). This data indicates that the P454-derived peptides exhibit an intrinsic propensity to fold in low dielectric constant environments, such as in lipid bilayers.

The addition of neutral membranes induced a variable increase in helical content of the different P454 peptides. This increase was further enhanced in the presence of negatively charged membranes, especially for the native and R12K peptides (Table S6 and Figure S6). This data indicates that the P454-derived peptides acquire secondary structures upon partitioning from the solution to the membrane, following a classical partitioning-folding coupling process [47,50].

**Figure 5:**
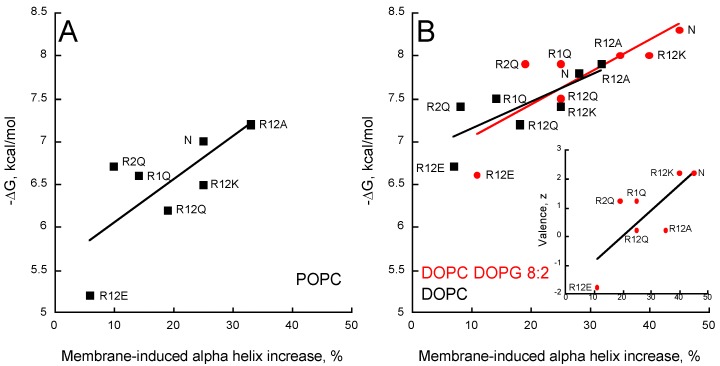
Partitioning-folding coupling of the P454-derived peptides. Helical content induced by the partition of peptides into POPC SUVs (**A**); DOPC SUVs (black squares) or DOPC/DOPG 8:2 SUVs (red circles) (**B**). For each peptide, the free energy of membrane partitioning is plotted as a function of the membrane-induced increase in helical content. ***Inset*** (**B**) Correlation between valence and membrane-induced helical content in the presence of DOPC/DOPG 8:2 SUVs. S.D. are ±0.1 kcal/mol.

The increase in alpha-helical content of the different peptides in neutral membranes (POPC and DOPC) nicely correlates with the helix propensity scale of amino acids A, R, K, Q, and E (0, 0.21, 0.26, 0.39, and 0.40 kcal/mol, respectively, see Table 3 from [51]). Figure 5 shows that the helical content increases in the presence of membrane as a function of the free energy of partition for all membrane lipid compositions. A similar trend is observed between the membrane-induced helical content and the peptide valence (inset Figure 5B). Interestingly, R2Q partly deviates from this trend, suggesting that R474 significantly contributes to the formation of an α-helix upon membrane partitioning, whatever the lipid composition (Figure 5 and inset Figure 5B). Finally, as observed above for partition from solution to membrane, no correlations were observed between the hydrophobic moment, the mean hydrophobicity, and the membrane-induced increase in helical content (Figure S7), indicating that the ability of the peptides to form a helix in the membrane is not directly related to their amphipathic character.

### 2.7. Membrane Permeabilization

**Figure 6:**
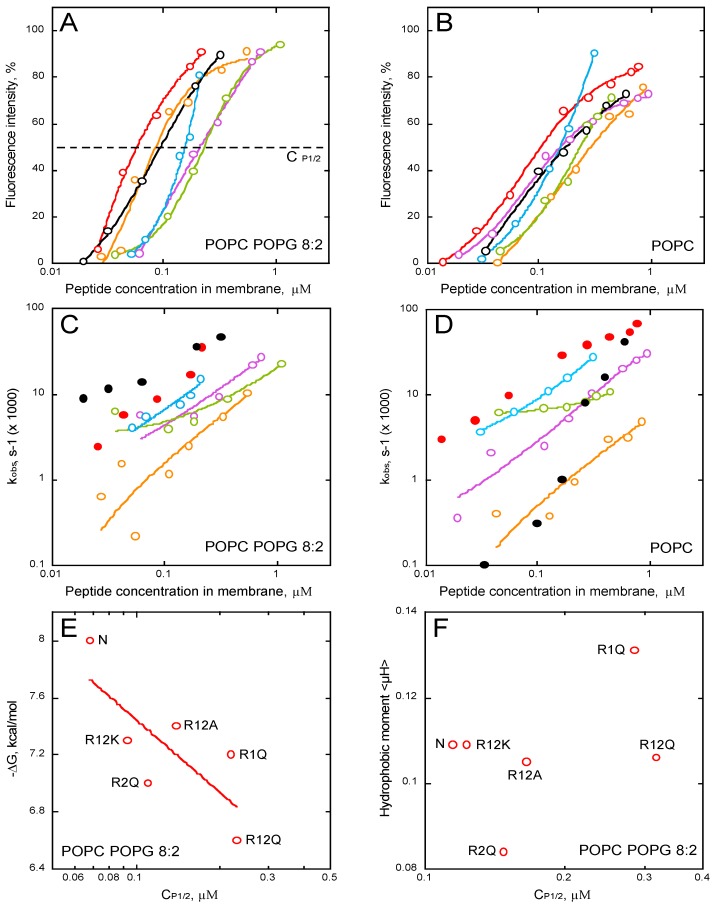
Membrane permeabilization by P454-derived peptides. Fluorescence intensities and observed constant rates, k_obs_, of P454 (open and close red symbols), R1Q (purple), R2Q (orange), R12Q (green), R12A (blue), and R12K (open and close black symbols) in the presence of POPC/POPG 8:2 (**A**,**C**) or POPC (**B**,**D**) LUVs, are shown as a function of the concentration of membrane-bound peptides. (**E**) ΔG values as a function of the peptide concentration required to induce half recovery of ANTS fluorescence, C_P1/2_, for the P454-derived peptides in POPC/POPG 8:2 LUVs. (**F**) Hydrophobic moment <μH> as a function of C_P1/2_ in the presence of POPC/POPG LUVs. The LUVs are at a final lipid concentration of 0.45 mM. Raw data of permeabilization traces are shown in Figure S8.

We studied membrane permeabilization induced by the P454 series by measuring the fluorescence recovery of ANTS upon vesicle efflux and dissociation from its quencher DPX. The kinetic traces are shown in supporting information (Figure S8). We then extracted the intensities of fluorescence recovery (Figure 6A,B) and the observed rate constants, k_obs_, (Figure 6C,D), for each condition, i.e., in the presence of LUVs composed of POPC (Figure 6B,D) and POPC/POPG 8:2 (Figure 6A,C). The intensity and k_obs_ values are plotted as a function of the concentrations of the peptide partitioned into the membrane (i.e., taking into account the partition coefficient K_X_ (see Methods)).

The results clearly show that all P454 peptides, except R12E, are able to permeabilize anionic and neutral membranes in a concentration-dependent manner; yet, permeabilization intensities and k_obs_ values are both affected by the arginine substitutions (Figure 6A–D). The peptides that most efficiently permeabilize anionic membranes are the native P454, R12K, and R2Q, while a single arginine substitution in R1Q strongly affects P454 activity (Figure 6A, in purple). The concentrations of membrane-bound peptides required to induce 50% of maximal ANTS fluorescence recovery, C_P1/2_, are extracted from Figure 6A. The free energy of binding is then plotted as function of C_P1/2_ (Figure 6E). Although the C_P1/2_ values generally correlate with the free energy of membrane partitioning, some peptide variants somehow diverge from this general tendency, most particularly R1Q. Indeed, although R1Q exhibits a rather high affinity for membranes (with a ∆G value in between those of R12K and R2Q; panel 6E, left part), its C_P1/2_ is relatively high, indicating that R1Q strongly partitions into membrane but is poorly efficient to destabilize lipid bilayers. Interestingly, R1Q exhibits the highest hydrophobic moment amongst the peptide series (Figure 6F) and acquires significant helical structures in membrane (Figure 5), suggesting that its weak permeabilization activity is not due to a loss of amphipathic property, nor helical content. Taken together, these results indicate that the arginine R461 of P454 specifically contributes to the anionic membrane permeabilization.

Interestingly, while the permeabilization rate constants of R12K are higher than those of WT P454 when tested on anionic membranes, they are strongly concentration-dependent when assayed on neutral membranes (Figure 6C,D, black circles). This suggests that lysines are able to induce a fast permeabilization in the presence of anionic lipids, while in the presence of neutral lipids, lysine residues are characterized by a significantly slower permeabilization than arginine ones (Figure 6D, red circles for WT P454). Although both arginine and lysine residues are positively charged, this data highlights the specific contribution of arginine side chains to membrane permeabilization (see discussion). 

## 3. Discussion

The CyaA toxin is one of the main virulent factors produced by *Bordetella pertussis*, the bacterium responsible for whooping cough. It invades eukaryotic target cells through an original mechanism that involves a direct translocation of its AC domain across the plasma membrane. However, the conformational states and the thermodynamics of AC membrane translocation are still poorly understood. We previously identified a “translocating region”, TR (see Figure 1A), which is essential for the transport of the AC domain into the cytoplasm of eukaryotic cells [10]. Indeed, deletion of this TR region selectively abolishes AC translocation across the membrane, while the cell binding and pore-forming (hemolysis) activities of the toxin are not affected. We then identified a shorter sequence, P454 (residues 454–484), which exhibits membrane-active properties, i.e., is able to both partition into and permeabilize membranes [11]. We proposed that once CyaA is inserted into the target cell membrane, this region might locally destabilize the lipid bilayer integrity, and hence, lower the energy required to translocate AC across the plasma membrane. Recently, Masin et al. showed that negatively charged residues (Asp and Glu) from TR are involved in modulating the pore-forming activity of CyaA, while mutations of positively charged residues within TR affect AC translocation [30].

Here, we further explored the structural and molecular properties of P454, the unique amphitropic peptide able to induce membrane permeabilization in the series we investigated (see below). We first characterized the effect of lipid composition on its membrane partitioning. We showed that P454 membrane interaction is favored by anionic lipid headgroups, as previously reported [11], and by acyl chain fluidity promoting liquid-disordered state (DO- vs. PO-acyl chains), while inverted hexagonal structures induced by cholesterol and PE lipids, promoting negative membrane curvature, disfavor P454 membrane interactions (Figure 3). Hence, membrane interaction of P454 is tightly regulated by the charge of the lipid head group and the physical properties of the lipid bilayer. P454 thus displays the classical properties of antimicrobial peptides (AMP) that undergo typical partition-folding coupling and shows greater affinity for negatively charged membranes than for neutral membranes. Moreover, lipid packing and order/rigidity—as induced by acyl chain saturation or cholesterol—decrease AMP membrane partitioning and permeabilization (see for instance [52] for a review), as observed here with P454 (Figure 3 and Figure 4). 

We then investigated the contributions of the arginine residues (R461 and R474) of P454 to membrane partitioning, folding, and permeabilization. P454 peptide variants were synthetized, in which R461 (called R1) and R474 (called R2) were substituted by various amino acids (A, K, Q, and E). Our results show that both arginine residues contribute, albeit differently, to the membrane-binding and permeabilization properties of P454. Our data provides direct evidence that arginine residues, compared to other amino acids, are appropriated for interactions with both neutral and anionic lipids, helical structure formation in membranes, and local destabilization of lipid bilayers. We found that membrane insertion of P454 involves both electrostatic interactions and hydrophobic forces. On the one hand, the mean net charge of the peptides (R1Q, R2Q, R12Q, R12K, R12A, and R12E) correlates with their membrane partitioning and secondary structure formation (Figure 4 and Figure 5). On the other, the efficient membrane partitioning of R12A, a P454 peptide with neutral charge, indicates that hydrophobic forces also significantly contribute to the peptide binding to lipid bilayer. 

To further investigate the role of the guanidinium group of arginine in P454 activity, the two arginines, R461 and R474, were substituted by lysine residues in peptide R12K. Although, R12K has the same positive charge as P454, it shows weaker membrane binding than P454 with all types of lipid vesicles (Figure 4 and Table S5). Moreover, R12K is less efficient at permeabilizing neutral membranes than the native P454 peptide (Figure 6D). The higher free energy value of membrane partition measured with the native P454 peptide, as compared to R12K, highlights the contribution of the interactions established between the arginine side chains and the surrounding phospholipids. The guanidinium group from arginine forms a guanidinium-phosphate complex stabilized by a combination of bidentate hydrogen-bonding and attractive electrostatic interactions [53]. Bidentate guanidinium-phosphate complexes can be stably established between one arginine and several phospholipids, while complexes between lysine residues and lipids are unstable [54]. It has been shown by NMR that lysine side chains, unlike those of arginine, do not form long-lasting hydrogen bonds with lipid phosphates [53,55], indicating that the amino group of lysine is engaged in weaker interactions with the lipid phosphates than the guanidinium group of arginine [56]. Finally, arginine side chains are more rigid and hydrophobic than the mobile, disordered, and solvated lysine side chains. These properties of arginine side chains, i.e., aliphatic and positively charged at neutral pH, favor the location of its methyl groups in the hydrophobic core of the lipid acyl chains, and the guanidinium groups strongly bind to phosphates of lipid headgroups. 

It has been shown that the network of interactions between arginine guanidinium groups and lipid phosphate groups induces distortion of the lipid bilayer [48,53,57,58], favoring membrane permeabilization, as observed with many AMPs that disrupt membrane integrity. Membrane insertion of such peptides induces a strong perturbation of lipid packing, favoring mixing of the acyl chains from the hydrophobic core with the polar groups from the lipid headgroups [59,60,61]. This local disorganization of the hydrophobic core and interfacial region allows the flux of molecules through the membrane. The arginine-induced membrane deformation and subsequent permeabilization is illustrated here by the comparison of the observed rate constants of P454 and R12K: in neutral membranes, WT P454, with its two native arginine residues, is able to induce a fast permeabilization at low peptide concentration while R12K requires high concentrations to speed up membrane permeabilization (Figure 6D). This highlights the strong impact on membrane binding and permeabilization of the guanidinium group of arginine as compared to the amino group of the lysine side chain. 

A comparative analysis of R1Q and R2Q peptides revealed that the two arginine residues distinctly contribute to P454 interaction with membranes: R474 is primarily involved in helix formation upon membrane association, while R461 is important for membrane permeabilization. These results suggest a model in which the C-terminal part of P454 forms a helical structure in the membrane, while the N-terminal part locally destabilizes the lipid bilayer. The corresponding sequence within the full-length CyaA toxin may likely act similarly on the target cell membranes. Once the hydrophobic and acylation regions are stably inserted into the membrane, the C-terminal region of P454, containing R474, might insert into the membrane and allow its N-terminal part, containing R461, to locally disrupt the lipid bilayer. This local membrane deformation could lead to the mixing of hydrophobic core and lipid headgroups, and thus help to decrease the energy required to translocate the N-terminal catalytic domain across the plasma membrane. In agreement with this, the protein CyaA_R461A_, in which R461 was substituted by an alanine residue, exhibited a significant reduction of AC translocation compared to the WT CyaA, although toxin-binding to the cells was not affected (see Figure 3 in Masin et al. [30]). The reduced level of AC translocation measured with CyaA_R461A_ may result from a lower membrane disruption capacity of R461, as evidenced by the permeabilization results obtained with R1Q (Figure 6E).

Finally, beyond the 454–484 segment from TR and the hydrophobic helical hairpins located in the hydrophobic region (500–750), we were interested in identifying other amphitropic helices that might be able to bind to membranes and destabilize lipid bilayers, and could thus contribute to the translocation process of CyaA. With this in mind, we have investigated the membrane-interacting properties of several short amphipathic segments from CyaA. They were selected according to their propensity to form helical structures, their local hydrophobicity, and their hydrophobic moment. Synthetic peptides modeled from these selected sequences were synthesized, and their abilities to partition from solution to membrane and to permeabilize membrane were analyzed by a combination of molecular dynamics, fluorescence, and circular dichroism experiments. We showed that several of these peptides exhibit membrane partitioning and secondary structure formation. However, none of them were able to permeabilize membranes (Figure 1, Figure 2, Figures S1 and S2). These different peptidic segments thus do not participate directly in the cell membrane destabilization, but they may contribute to appropriately docking the CyaA polypeptide chain on the surface of the plasma membrane of target cells in order to facilitate the transport of the catalytic domain across the lipid bilayer. This is particularly the case for P233, which could maintain the AC domain in close proximity to the membrane before translocation.

## 4. Conclusions

Many bacterial toxins need to invade eukaryotic cells to access an intracellular molecular target. Few of them carry their own translocation systems to deliver their catalytic domains into cell cytoplasm. This is the case, for example, of the diphtheria toxin, botulinum toxins, lethal toxin, and CyaA toxin [62,63,64,65,66]. These toxins are amphitropic, i.e., they are soluble proteins that are able to insert into membranes and translocate their catalytic domains across target membranes [67]. Interestingly, besides their hydrophobic helices, which likely favor membrane insertion of the toxins into the hydrophobic core of lipid bilayers, these proteins possess arginine rich, amphitropic segments able to locally destabilize lipid bilayers, such as TH1 to TH4 in the diphtheria toxin [14,68] and the translocation region of CyaA [10,11]. These regions thus act as membrane permeabilizing peptides embarked into bacterial toxins and are actively involved in the translocation process by locally disrupting the membrane integrity, a process that decreases the energy required to transport catalytic domains into target cells.

## 5. Materials and Methods

### 5.1. Reagents

1-palmitoyl-2-oleoyl-*sn*-glycero-3-phosphocholine (POPC, reference 850457C), 1-palmitoyl-2-oleoyl-*sn*-glycero-3-[phospho-*rac*-(1-glycerol)] (POPG, reference 840457C), 1,2-dioleoyl-*sn*-glycero-3-phosphocholine (DOPC, reference 850375C), 1,2-dioleoyl-*sn*-glycero-3-[phospho-*rac*-(1-glycerol)] (DOPG, reference 840475C), 1,2-dioleoyl-*sn*-glycero-3-phosphoethanolamine (DOPE, reference 850725C), and cholesterol (Chol, reference 700000P) were from Avanti Polar Lipids (Alabaster, AL, USA). All experiments were performed in 20 mM HEPES, 150 mM NaCl, pH 7.4 (buffer A). ANTS (A-350, 8-aminonapthalene-1,3,6 trisulfonic acid), and DPX (X-1525, p-xylene-bis-pyridinium bromide) were purchased from Molecular Probes (Eugene, OR, USA).

### 5.2. Peptides

All peptides were produced and purified by Genosphere Biotechnologies (Paris, France) and their purity, as their molecular weight, were controlled by HPLC and MALDI. All peptides were capped on the N-terminus with an acetyl group and on the C-terminus with an amide group. We studied 11 CyaA-derived peptides: P19, P89, P233, P414, P454, P495, P509, P626, P717, P737, and P777 peptides, as well as 6 P454-derived peptides (R1Q, R2Q, R12Q, R12A, R12K, and R12E). The peptides were selected according to their propensity to form helical structures, their local hydrophobicity, and their hydrophobic moment (Figure S1).

- The P19 peptide corresponds to the B-helix in AC, i.e., residues 19 to 35 of CyaA (IPAAVLDGIKAVAKEKNW), and a tryptophan was added at its C-terminal extremity (underlined).
- The P89 peptide corresponds to the D-helix in AC, i.e., residues 89 to 107 (APEVIARADNDVNSSLAHGW), with a tryptophan added at its C-terminal extremity (underlined).
- The P233 peptide corresponds to the H-helix in AC, i.e., residues 233 to 254 of CyaA (LDRERIDLLWKIARAGARSAVG), and contains the native tryptophan W242.
- The P414 peptide corresponds to residues 414 to 440 of CyaA (SW SLGEVSD- MAAVEAAELEMTRQVLHA), in which Phe-415 was substituted by a tryptophan residue (underlined).
- The P454 peptide corresponds to residues 454 to 484 of CyaA (ASAHWGQRALQGAQAVAAAQRLVHAIALMTQ) and contains the single native tryptophan W458. Several P454-derived peptides were also synthetized, in which the two arginine residues R461 (hereafter referred to as R1) or R474 (referred to as R2), or both of them (referred to R12) were substituted by either lysines (R12K), glutamines (R1Q, R2Q and R12Q), alanines (R12A), or glutamates (R12E).
- The P495 peptide corresponds to residues 495 to 524 of CyaA (QEAASLSAAVFGLGEASSAVAETVSGFFRGW), and a tryptophan was added at its C-terminal extremity (underlined).
- The P509 peptide is a shorter peptide from P495, corresponding to residues 509 to 524 of CyaA (EASSAVAETVSGFFRGW), and a tryptophan was added at its C-terminal extremity (underlined).
- The P626 peptide corresponds to residues 626 to 647 of CyaA (LVQQSHYADQLDKLAQESSAYGW), and a tryptophan was added at its C-terminal extremity (underlined).
- The P717 peptide corresponds to residues 717 to 733 of CyaA (IIEKLANDYARKIDELGW), and a tryptophan was added at its C-terminal extremity (underlined).
- The P737 peptide corresponds to residues 737 to 771 of CyaA (AYFEKNLQARHEQLANSDGLRKMLADLQAGWNASS) and contains the single native tryptophan W767.
- Finally, the P777 peptide corresponds to residues 777 to 793 of CyaA (TTEISKSALELAAITGNW) and a tryptophan was added at its C-terminal extremity (underlined).

The physical chemistry of these peptides is described in Tables S1 and S2.

### 5.3. Lipid Vesicles Preparation

Small unilamellar vesicles (SUVs) were prepared at a lipid concentration of 10 mM from POPC and POPG at a molar ratio of 10:0 or 8:2 or from DOPC, DOPE, DOPG, Chol at molar ratio 10:0:0:0, 8:0:2:0, 7:3:0:0, 7:0:0:3, 6:3:0:1, and 4:3:2:1 in buffer A, as previously described [69]. Briefly, SUVs were prepared by reverse-phase evaporation and passed through polycarbonate filters 0.8-, 0.4-, and 0.2-μm (Merck Millipore, Darmstadt, Germany). Vesicles were sonicated using a sonicator tip and their hydrodynamic diameters, dispersity, and charge were checked by dynamic light scattering (DLS) and electrophoretic mobility using a NanoZS instrument (Malvern Instruments, Orsay, France).

### 5.4. Tryptophan Fluorescence Titrations

Fluorescence titrations were performed with a FP-8200 Jasco spectrofluorimeter, equipped with a Peltier-thermostated ETC-272T (25 °C). A bandwidth of 5 nm was used for both excitation and emission beams. Fluorescence experiments were carried out in a 109.004F-QS cuvette (Hellma, France) with constant stirring. Fluorescence emission was recorded between 300 and 400 nm at a scan rate of 200 nm/min, with an excitation wavelength of 280 nm. Fluorescence emission spectra were corrected for SUV light scattering. Maximum emission wavelength (λ_max_) and fluorescence intensity ratio at 330 nm over 370 nm (P454-derived peptides) or at 340 nm over 380 nm (CyaA-derived peptides) were used to report peptide-membrane interaction and to measure the partition coefficient K_X_.

### 5.5. Peptide Partition from Solution to Membranes

The partition coefficient K_X_ is defined as the ratio of protein concentrations in the lipid and in buffer phases [10,11,47]. It is expressed as follows:

$K_{X}=\frac{P_{L}}{P_{W}}\cdot \frac{\left[W\right]}{\left[L\right]}$, providing $\frac{P_{W}}{P_{L}}=\frac{\left[W\right]}{K_{X}[L]}$ , where *P_W_* is the concentration of peptide in solution, *P_L_* the concentration of peptides partitioning into lipid membrane, [*W*] represents the concentration of water, and [*L*] the lipid concentration.

The fraction of peptide partitioned into the membrane phase, *f_PL_*, is equal to:

$$f_{PL}=\frac{P_{L}}{P_{T}}=\frac{P_{L}}{P_{L}+P_{W}}=\frac{1}{(P_{W}/P_{L})+1}=\frac{1}{\left(\frac{\left[W\right]}{K_{X}[L]}\right)+1}$$

The partition coefficient is related to the apparent dissociation constant as follows: *K_X_* * *K_D_* = [*W*] with *K_D_* * *P_L_* = *P_W_* * [*L*]. The equation *f_PL_* is fitted to the experimental data using the KaleidaGraph software, providing K_X_. The free energy corresponding to the transfer of the peptide from the aqueous phase to the lipid phase is given by the equation $\Delta G=-RT\ln\left(K_{X}\right)$, where Δ*G* is the free energy in kcal/mol, *R* the gas constant (*R* = 1.98 × 10^−3^ kcal/mol/K), and *T* the temperature in Kelvin.

### 5.6. Circular Dichroism

Synchrotron radiation circular dichroism (SRCD) experiments were carried out on the DISCO beamline of Synchrotron SOLEIL (Gif-sur-Yvette, France). SRCD spectra were recorded at 25 °C with an integration time of 1.2 s and a bandwidth of 1 nm with a resolution of 1 nm. Each far-UV spectrum represents the average of three individual scans. QS cells (Hellma, France) with a path length of 100 μm were used to record SRCD signal in the far-UV (from 190 to 250 nm). Peptides were tested at 100 μM in buffer A and in the presence of 20% of trifluoroethanol (TFE). Peptides were incubated in the presence of SUVs (POPC, DOPC, or DOPC/DOPG 8:2) at final concentration of 2 mM lipids. Buffer A, in the absence or presence of SUVs, was used as blank and subtracted from far-UV CD spectra of peptides. Secondary structure content was estimated using Bestsel [49]. 

### 5.7. Membrane Permeabilization

ANTS (fluorophore probe) and DPX (quencher) were encapsulated into large unilamellar vesicles (LUVs) to monitor membrane permeabilization induced by the peptides. The LUVs were prepared at a concentration of 10 mM lipid at the following molar ratio DOPC/DOPE/DOPG/Chol 4:3:2:1, POPC/POPG 8:2, or POPC containing 20 mM ANTS and 60 mM DPX. The multilamellar vesicle suspension was passed through 0.4- and 0.2-μm polycarbonate filters to produce LUVs of 200 ± 30 nm in diameter, as measured by DLS. The unencapsulated probes were removed by gel filtration through a Sephadex G-25 column 5 mL (GE Healthcare Life Sciences, Pittsburgh, PA, USA). For permeabilization assays, LUVs were incubated in buffer at 0.45 mM lipids at 25 °C in a 101-QS cuvette (Hellma, France) and under constant stirring. The excitation wavelength was set to 390 nm and the emission of ANTS was continuously measured at 515 nm. A single exponential equation was fitted to kinetic traces to extract fluorescence intensity and observed rate constants, k_obs_. The percentage of fluorescence intensity was calculated using the maximum intensity measured after complete disruption of the vesicles with addition of 0.12% (2 mM) of Triton. The concentration of membrane-bound peptide required to induce half-maximal ANTS fluorescence recovery (C_P1/2_) is extracted from the plot of permeabilization intensity as a function of peptide concentration (Figure 6A). 

### 5.8. Molecular Dynamics

The coarse-grained simulations were carried out with Gromacs 4.5.5 [70] using the Martini force field [71,72,73] (md.chem.rug.nl). The coarse-grained model of the P233, P454, P509, and P717 were directly derived from the all-atom NMR models. Firstly, 1000 lipids were randomly mixed with water and charged beads mimicking counter-ions. Two ratios of DOPC/DOPG/Cholesterol were used: 8:1:1 and 9:0:1 in order to study the influence of negatively charged lipids. The lipid-water mixture was equilibrated over 20 ns using a 20 fs time step. The temperature was maintained to 323.15 K using velocity rescaling [74] and a coupling constant of 1 ps. The pressure was maintained to 1 bar using a semi-isotropic Berendsen barostat [75], a coupling constant of 2 ps, and a compressibility of 3e^−4^ bar^−1^. Van der Waals interaction was cut off at 12 Å with a shift function applied beyond 9 Å. Electrostatics were cut-off at 14 Å with long range electrostatics treated by the particle mesh Ewald method [76,77] using a grid spacing of approximately 1 Å. The dielectric constant was set to 15. The two systems were simulated over 0.5 μs, i.e., until a lipid bilayer at equilibrium was obtained. It should be noted that bilayers were self-assembled in approximately 0.1 µs. Secondly, the resulting bilayers were extracted and the peptides P233, P454, P509, and P717 were placed at approximately 4.8 nm from the bilayer center, i.e., 0.8 nm above the bilayer surface. The systems were fully hydrated and neutralized with charged beads. The final size of the lipid-peptide systems was approximately 16 × 20 × 23.5 nm^3^. Each system was equilibrated over 20 ns in conditions identical to the lipid-water mixture, then simulated over 1 μs. Visualization of the trajectories was done with VMD 1.9.1 [78]. Their analysis was carried out with Gromacs 4.5.5 [70], R 2.14.1 [79], and the ggplot2 package [80]. 

## Supplementary Materials

The following are available online at www.mdpi.com/2072-6651/9/11/369/s1: Figure S1: Hydrophobic moment <μH> as function of the mean hydrophobicity <H> of the CyaA-derived peptides; Figure S2: Far-UV circular dichroism spectra of the CyaA-derived peptides; Figure S3: Partition of CyaA-derived peptides followed by tryptophan intrinsic fluorescence; Figure S4: Free energy of partition of peptides in POPC/POPG membranes as a function of mean hydrophobicity <H> or hydrophobic moment <μH>; Figure S5: Synchrotron radiation far-UV circular dichroism of P454-derived peptides; Figure S6: Helical content of the P454-derived peptides in various environments; Figure S7: Membrane-induced helical content in the presence of DOPC/DOPG SUVs as a function of mean hydrophobicity <H> or hydrophobic moment <μH>; Figure S8: Membrane permeabilization induced by the P454-derived peptides; Table S1: Peptide names, number of residues, molecular weight, pK and charge at physiological pH of each peptide used in this study; Table S2: Maximum mean hydrophobicity <H> and hydrophobic moment <μH> of each peptide using their full sequence or a window of 12 to 18 amino acids; Table S3: Thermodynamics of peptide partitioning from solution to membrane; Table S4: Effect of lipid bilayer composition on P454 membrane partitioning; Table S5: Partition of P454-derived peptides from solution to membrane; Table S6: Estimation of the α helical content of peptides.
