# Supplementary material for: Membrane-Active Properties of an Amphitropic Peptide from the CyaA Toxin Translocation Region
Source: Toxins (Basel). 2017 Nov 14;9(11):369. doi: 10.3390/toxins9110369 (PMC5705984; doi:10.3390/toxins9110369)
Supplement: Supplementary file 1 [file toxins-09-00369-s001.pdf]

Supplementary Materials: Membrane-Active Properties of an Amphitropic Peptide from the CyaA Toxin Translocation Region

Alexis Voegele, Orso Subrini, Nicolas Sapay, Daniel Ladant and Alexandre Chenal

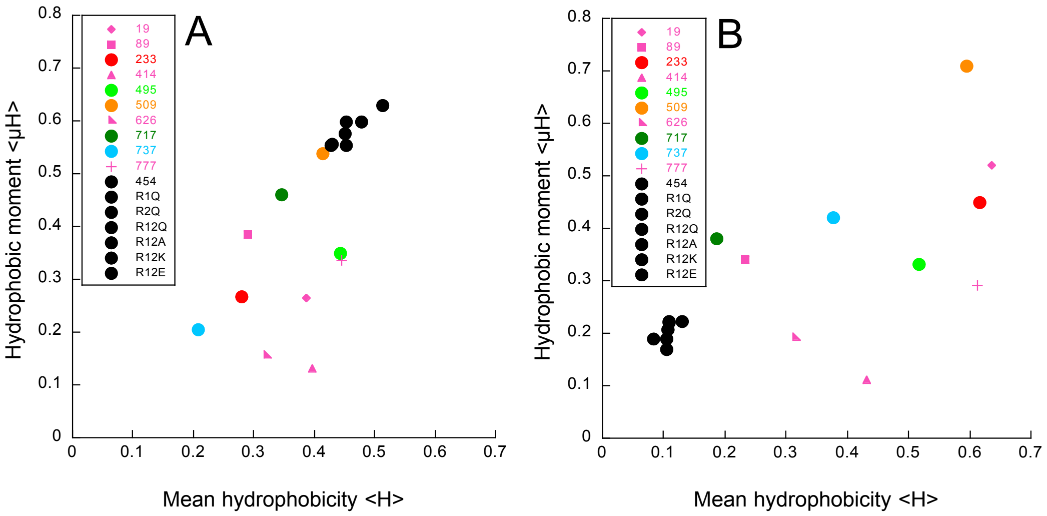

Figure S1. Hydrophobic moment <µH> as function of the mean hydrophobicity <H> of the CyaA-derived peptides. <H> and <µH> values were calculated with the Heliquest software using the sequences of the peptides (**A**) or using a window of 12 to 18 amino acids (**B**) (Table S2). Circles represent peptides interacting with membranes. Others symbols represent peptides that do not interact with membranes.

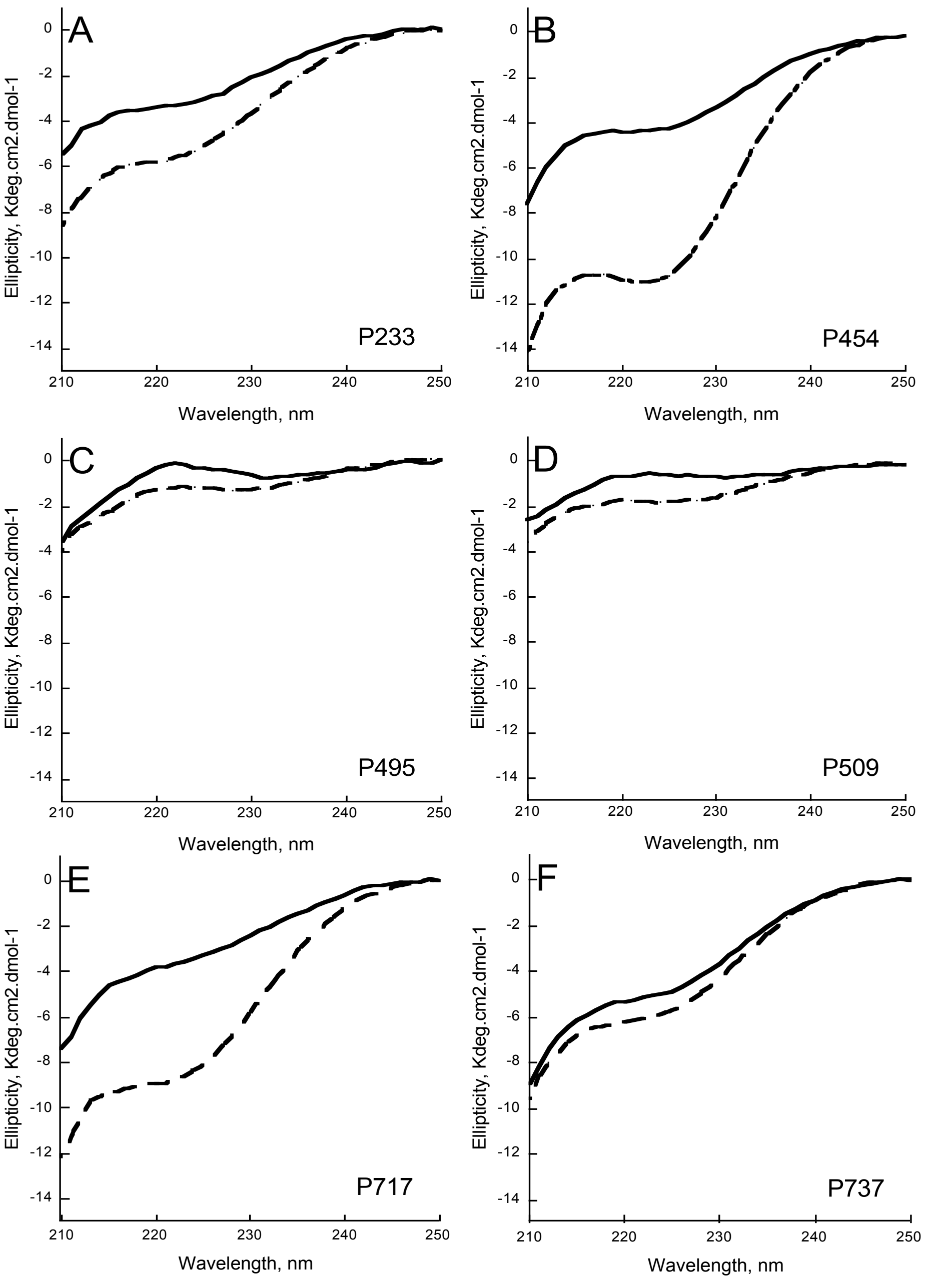

Figure S2. Far-UV circular dichroism spectra of the CyaA-derived peptides. CD spectra of P233 (**A**), P454 (**B**), P495 (**C**), P509 (**D**), P717 (**E**) and P737 (**F**) peptides at 40 μM in solution (solid lines) or in the presence of SUVs DOPC/DOPE/DOPG/Chol 4:3:2:1 at 1 mM lipids (dashed lines). CD spectra were recorded on an Aviv circular dichroism spectropolarimeter model 215. Briefly, CD measurements were carried out at a scan rate of 0.5 nm/sec (step: 0.5 nm and integration time: 1 sec) with a time constant of 100 msec and a bandwidth of 1 nm. Rectangular quartz Suprasil cells of 1 mm path lengths (110.QS, Hellma) were used for measuring CD activity in the far-UV region. Each far-UV CD spectrum represents the average of 5 scans.

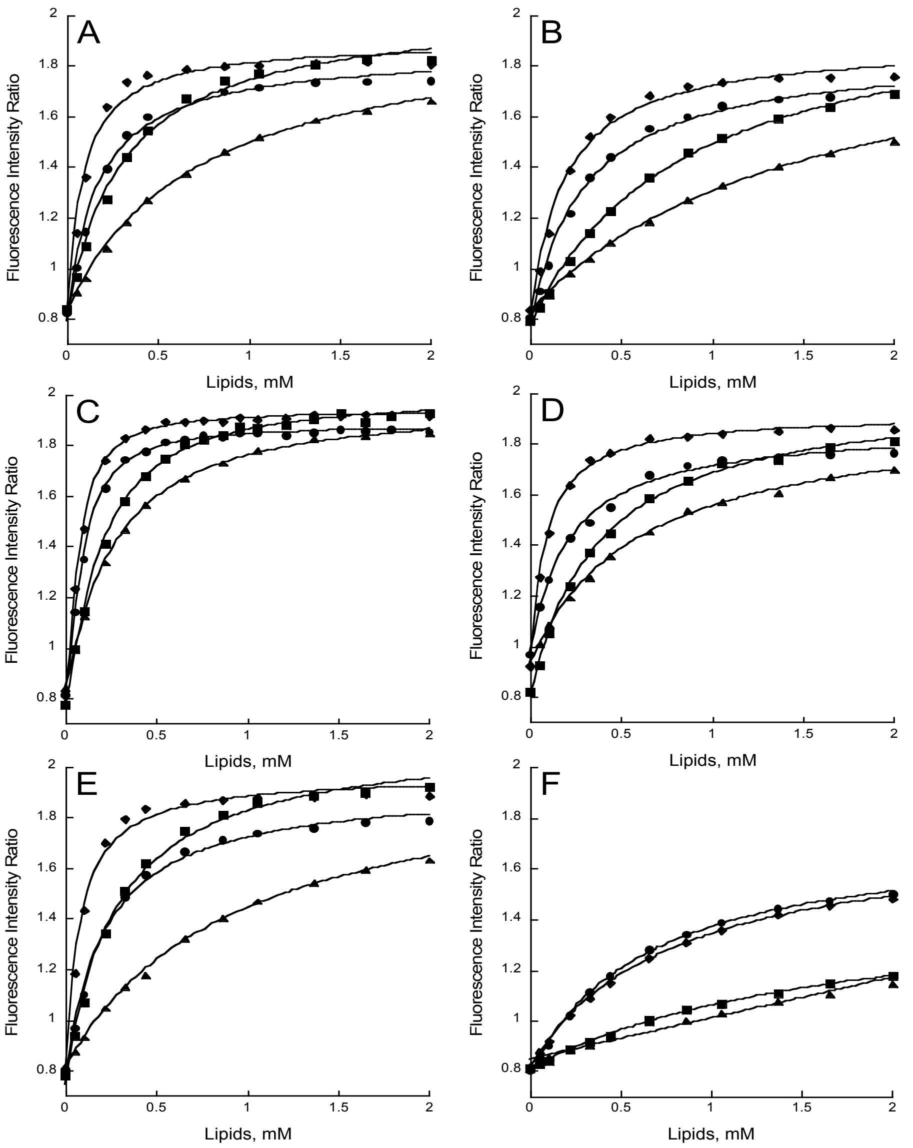

Figure S3. Partition of CyaA-derived peptides followed by tryptophan intrinsic fluorescence. Ratio of fluorescence intensity at 330 nm over 370 nm (excitation at 280 nm) as a function of lipid concentration (0 to 2 mM) for P454 R1Q (**A**), P454 R12Q (**B**), P454 R12A (**C**), P454 R2Q (**D**), P454 R12K (**E**) and P454 R12E (**F**) in POPC/POPG 8:2 (square), POPC (triangle), DOPC/DOPG 8:2 (diamond) or DOPC (circle) SUVs. The fitting procedure is described in Materials and Methods.

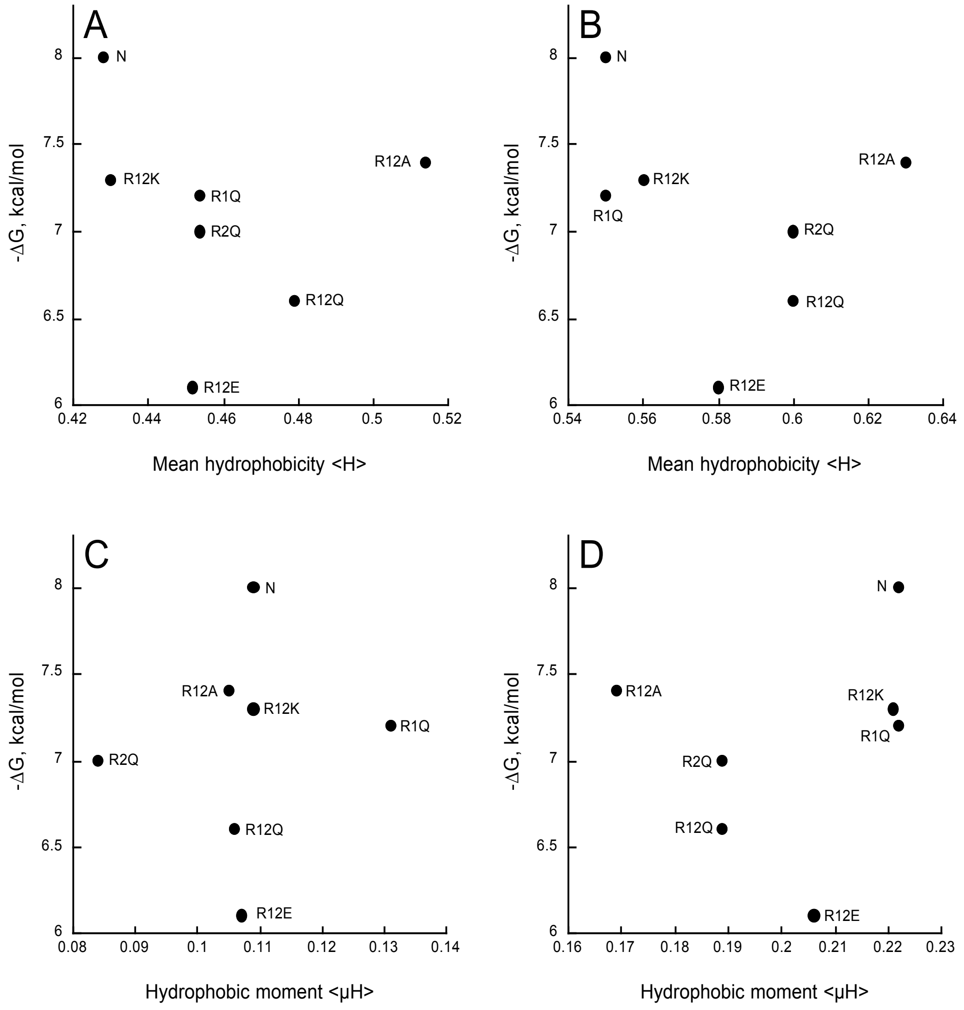

Figure S4. Free energy of partition of peptides in POPC/POPG membranes as a function of mean hydrophobicity <H> or hydrophobic moment <μH>. <H> and <μH> was extracted from Heliquest software for the sequences of the entire peptides (**A**, **C**) or for a window of 18 amino acids (**B**, **D**) corresponding to the C-terminal part of each peptide. These values are reported in Table S2.

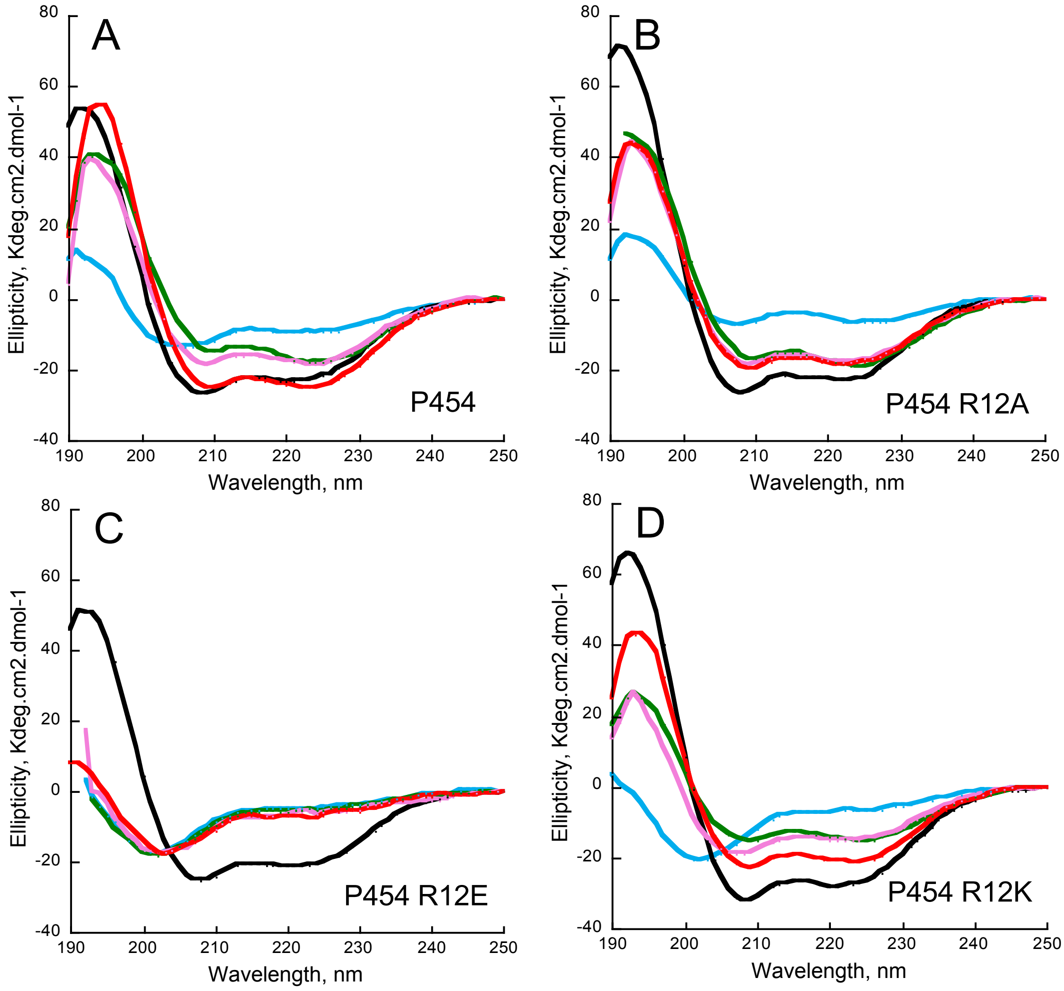

Figure S5. Synchrotron radiation far-UV circular dichroism of P454-derived peptides. The peptide concentration is 100 μM in buffer A at 25°C (blue), in the presence of 20% TFE (black) or in the presence of SUVs at 2 mM of POPC (green), DOPC/DOPG 8:2 (red) or DOPC (purple). Far-UV CD spectra of the following peptides are shown: P454 (**A**), P454 R12A (**B**), P454 R12E (**C**) and P454 R12K (**D**).

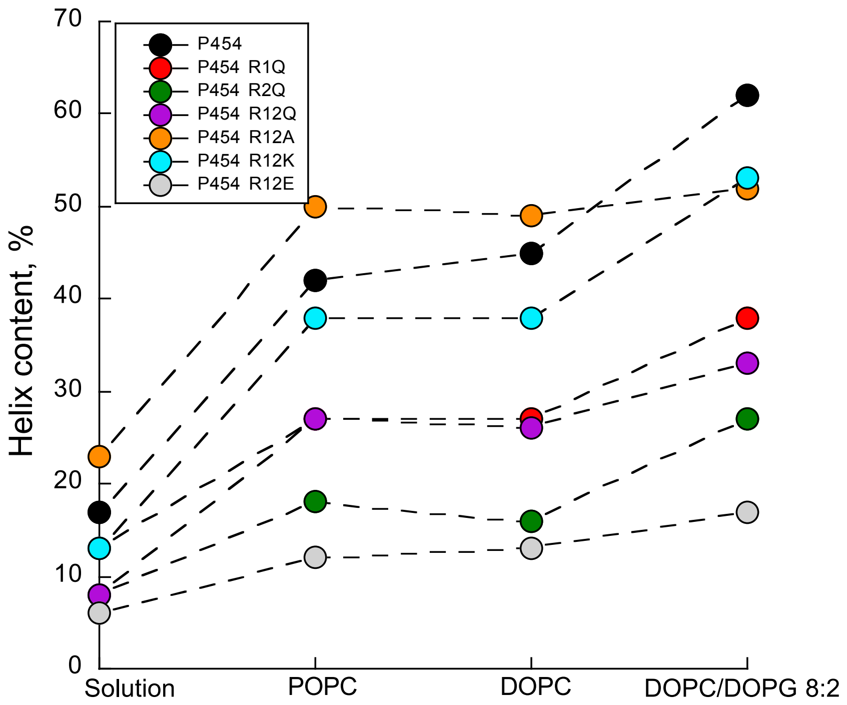

Figure S6. Helical content of the P454-derived peptides in various environments. The helical content of the peptides in solution, in the presence of POPC, DOPC and DOPC/DOPG 8:2 membranes is estimated using the BestSel software. The color code for the P454-derived peptides is the following: P454 (black), P454 R1Q (red), P454 R2Q (green), P454 R12Q (purple), P454 R12A (orange), P454 R12K (blue) and P454 R12E (grey). Bestsel estimations of the peptide helical contents are reported in Table S6.

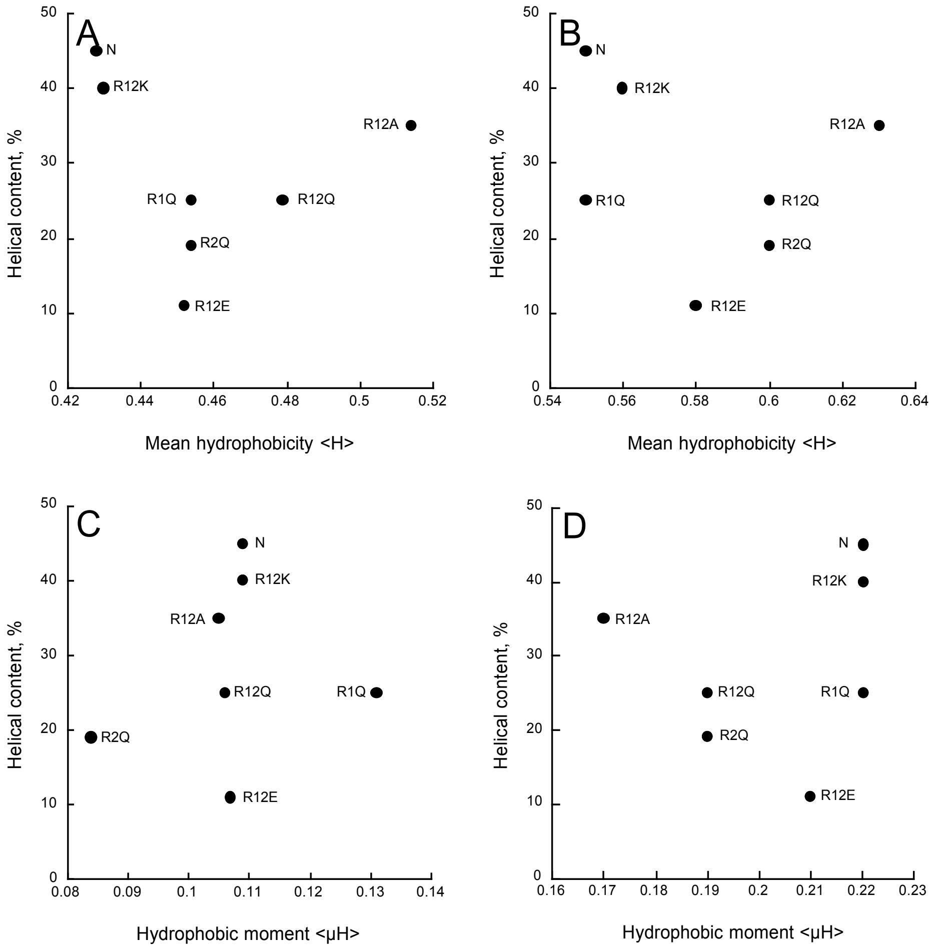

Figure S7. Membrane-induced helical content in the presence of DOPC/DOPG SUVs as a function of mean hydrophobicity <H> or hydrophobic moment <μH>. The values of <H> and <μH> were extracted from Heliquest software using the sequences of the peptides (**A**, **C**) or a window of 18 amino acids (**B**, **D**) corresponding to the C-terminal part of each peptide. These values are reported in Table S2.

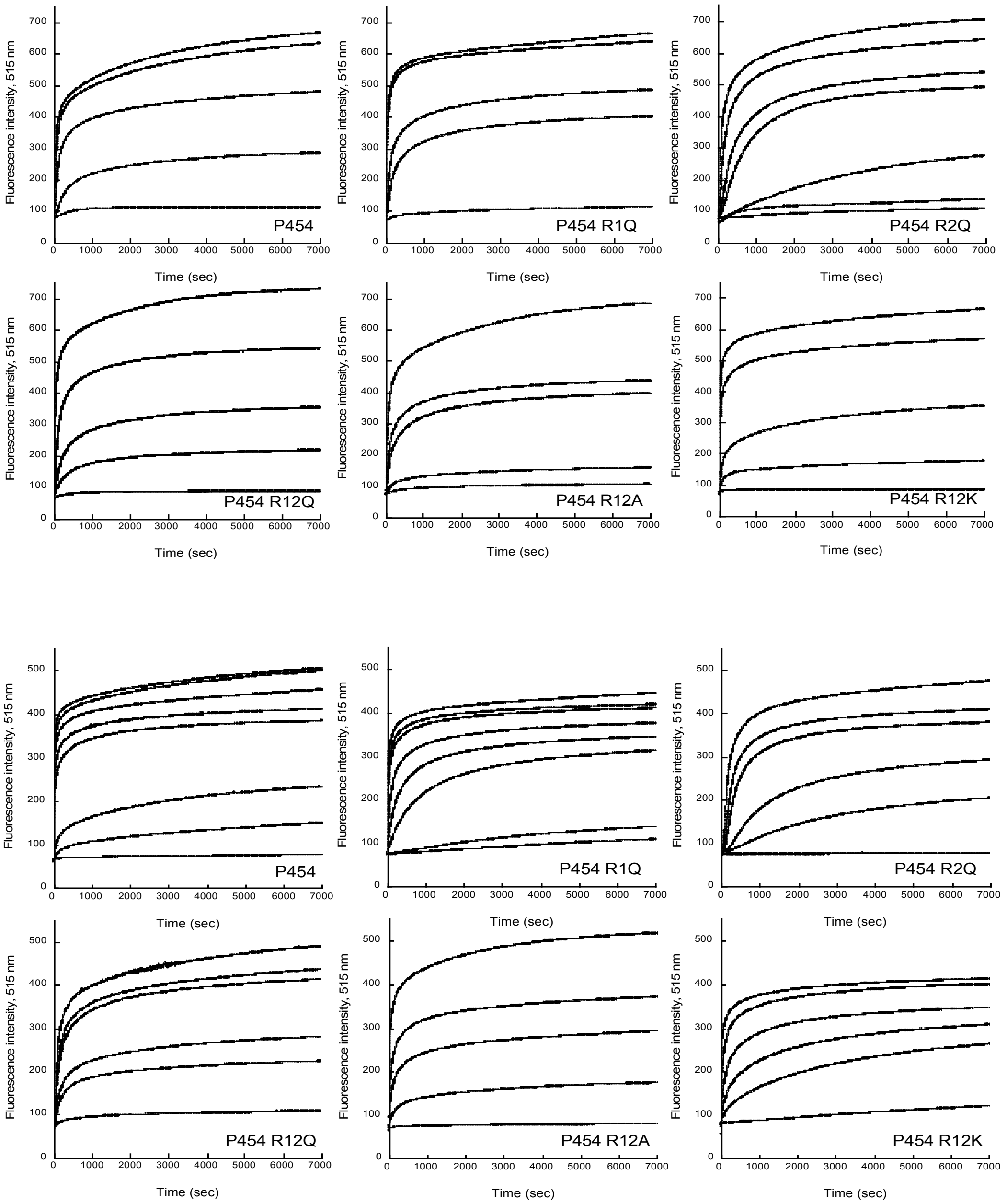

Figure S8. Membrane permeabilization induced by the P454-derived peptides. Top and bottom panels correspond to permeabilization assays performed in the presence of POPC/POPG 8:2 and POPC LUVs at 0.45 mM in solution, respectively. Each trace corresponds to a peptide at a given concentration (the peptide concentrations are reported in Figure 6). Peptide names are indicated in each panel.

Table S1. Peptide names, number of residues, molecular weight, pK and charge at physiological pH of each peptide used in this study.

Peptide	Number of residues	Molecular weight (g/mol)	pK	Charge at pH 7
P19	18	1 965	10	+1
P89	20	2 164	4.2	-2
P233	22	2 509	10.8	+2
P414	27	2 972	4	-4
P454	31	3 268	12	+2.2
P454 R1Q	31	3 240	10.9	+1
P454 R2Q	31	3 240	10.9	+1.2
P454 R12Q	31	3 211	8	+0.2
P454 R12A	31	3 097	8	+0.2
P454 R12K	31	3 212	10.7	+2.2
P454 R12E	31	3 214	5.1	-1.8
P495	31	3 101	4	-2
P509	17	1 842	4.3	-1
P626	23	2 678	4.7	-2
P717	18	2 188	4.8	-1
P737	35	3 975	7.7	0
P777	18	1 946	4.5	-1

Table S2. Maximum mean hydrophobicity <H> and hydrophobic moment <μH> of each peptide using their full sequence or a window of 12 to 18 amino acids. Values were extracted from Heliquest.

Peptide	Mean hydrophobicity <H> full peptide	Hydrophobic moment <μH> full peptide	Mean hydrophobicity <H> window of 12 to 18 amino acids	Hydrophobic moment <μH> window of 12 to 18 amino acids
P19	0.387	0.264	0.636	0.521
P89	0.29	0.385	0.233	0.341
P233	0.28	0.267	0.617	0.448
P414	0.397	0.132	0.431	0.112
P495	0.444	0.35	0.517	0.332
P509	0.415	0.537	0.596	0.71
P626	0.321	0.155	0.315	0.192
P717	0.346	0.46	0.187	0.381
P737	0.209	0.205	0.378	0.42
P777	0.445	0.336	0.613	0.292
P454	0.428	0.554	0.109	0.222
P454 R1Q	0.454	0.554	0.131	0.222
P454 R2Q	0.454	0.598	0.084	0.189
P454 R12Q	0.479	0.598	0.106	0.189
P454 R12A	0.514	0.628	0.105	0.169
P454 R12K	0.43	0.556	0.109	0.221
P454 R12E	0.452	0.575	0.107	0.206

Table S3. Thermodynamics of peptide partitioning from solution to membrane. SUVs were composed of DOPC/DOPE/DOPG/Chol at a ratio of 4:3:2:1. The P19, P89, P414, P626 and P777 peptides do not partition into membrane. *Secondary structure is inferred from both far-UV and MD results.

Peptide	K_X	ΔG, kcal/mol	Secondary structure*
P454	300 000	-7.4	α helix
P495	130 000	-6.9	non canonical
P509	80 000	-6.7	non canonical
P233	60 000	-6.5	α helix
P717	60 000	-6.5	α helix
P737	20 000	-5.8	α helix

Table S4. Effect of lipid bilayer composition on P454 membrane partitioning. All K_X and ΔG values were obtained for the WT P454 in the presence of membranes with various lipid compositions.

Membranes	K_X	ΔG, kcal/mol
DOPC/DOPG 8:2	1 200 000	-8.3
POPC/POPG 8:2	790 000	-8
DOPC	505 000	-7.8
DOPC/DOPE/DOPG/Chol 4:3:2:1	300 000	-7.4
POPC	150 000	-7
DOPC/DOPE 7:3	80 000	-6.7
DOPC/Chol 7:3	60 000	-6.5
DOPC/DOPE/Chol 6:3:1	40 000	-6.3

Table S5. Partition of P454-derived peptides from solution to membrane. The first value corresponds to the partition coefficient K_X and the second value to the free energy ΔG in kcal/mol.

Peptide	POPC/POPG 8:2	POPC	DOPC/DOPG 8:2	DOPC
P454	790 000 / -8	150 000 / -7	1 200 000 / -8.3	505 000 / -7.8
P454 R1Q	190 000 / -7.2	75 000 / -6.6	670 000 / -7.9	340 000 / -7.5
P454 R2Q	150 000 / -7	90 000 / -6.7	655 000 / -7.9	260 000 / -7.4
P454 R12Q	70 000 / -6.6	35 000 / -6.2	310 000 / -7.5	195 000 / -7.2
P454 R12A	275 000 / -7.4	195 000 / -7.2	795 000 / -8	610 000 / -7.9
P454 R12K	215 000 / -7.3	60 000 / -6.5	750 000 / -8	265 000 / -7.4
P454 R12E	30 000 / -6.1	7 000 / -5.2	70 000 / -6.6	85 000 / -6.7

Table S6. Estimation of the α helical content of peptides using Bestsel in solution, in TFE and in the presence of membranes of various lipid compositions. The last column reports the helical content predicted with the software SOPMA.

Peptide	% helix in solution	% helix in TFE 20%	% helix in POPC	% helix in DOPC	% helix in DOPC/ DOPG 8 :2	helical content (%) predicted by SOPMA
P454	17 ± 2	60 ± 2	42 ± 3	45 ± 5	62 ± 1	100
P454 R1Q	13 ± 1	41 ± 3	27 ± 1	27 ± 1	38 ± 2	80
P454 R2Q	8 ± 3	45 ± 3	18 ± 1	16 ± 1	27 ± 2	90
P454 R12Q	8 ± 4	50 ± 2	27 ± 2	26 ± 3	33 ± 3	80
P454 R12A	23 ± 2	61 ± 2	50 ± 3	49 ± 4	52 ± 2	80
P454 R12K	13 ± 2	72 ± 1	38 ± 1	38 ± 2	53 ± 3	80
P454 R12E	6 ± 1	55 ± 3	12 ± 3	13 ± 1	17 ± 2	80
